# Aspirin Eugenol Ester Modulates the Hypothalamus Transcriptome in Broilers Under High Stocking Density

**DOI:** 10.3390/ani15060823

**Published:** 2025-03-13

**Authors:** Xiaodie Zhao, Yi Zhang, Dongying Bai, Wenrui Zhen, Caifang Guo, Ziwei Wang, Penghui Ma, Xiqiang Ma, Xiaolin Xie, Koichi Ito, Bingkun Zhang, Yajun Yang, Jianyong Li, Yanbo Ma

**Affiliations:** 1 Department of Animal Physiology, College of Animal Science and Technology, Henan University of Science and Technology, Luoyang 471003, China; zhaoxiaodie@stu.haust.edu.cn (X.Z.); zhangyi439250@gmail.com (Y.Z.); zhenwenr@126.com (W.Z.); guocaifang@stu.haust.edu.cn (C.G.); wangziwei@stu.haust.edu.cn (Z.W.); mapenghui@stu.haust.edu.cn (P.M.); 2 Henan International Joint Laboratory of Animal Welfare and Health Breeding, College of Animal Science and Technology, Henan University of Science and Technology, Luoyang 471023, China; 3 Innovative Research Team of Livestock Intelligent Breeding and Equipment, Science & Technology Innovation Center for Completed Set Equipment, Longmen Laboratory, Luoyang 471023, China; maxiqiang@haust.edu.cn (X.M.); xiexiaolin@haust.edu.cn (X.X.); 4 Department of Food and Physiological Models, Graduate School of Agricultural and Life Sciences, The University of Tokyo, Tsukuba 319-0206, Japan; akoito@mail.ecc.u-tokyo.ac.jp; 5 State Key Laboratory of Animal Nutrition, Department of Animal Nutrition and Feed Science, College of Animal Science and Technology, China Agricultural University, Beijing 100193, China; bingkunzhang@126.com; 6 Key Lab of New Animal Drug of Gansu Province, Key Lab of Veterinary Pharmaceutical Development of Ministry of Agriculture and Rural Affairs, Lanzhou Institute of Husbandry and Pharmaceutical Science of Chinese Academy of Agricultural Sciences, Lanzhou 730046, China; yangyajun@caas.cn (Y.Y.); lijy1971@163.com (J.L.)

**Keywords:** high stocking density, broiler, aspirin eugenol ester, hypothalamus, RNA-seq, transcriptome

## Abstract

Broilers grown in a high-density (HD) stocking environment may experience intense competition that may adversely affect their growth relative to animals reared at a normal density (ND). The growth performance of HD broilers is increased by aspirin eugenol ester (AEE), although the mechanism by which this compound modulates hypothalamus-regulated feeding behavior is unclear. The aims of this study were to determine the effects of including AEE in the basal diet on the hypothalamic transcriptome and to examine in parallel the impact of these modifications on broiler production performance in HD conditions. Three hundred sixty one-day-old male Arbor Acres broilers were randomly divided into four groups: an ND group (14 broilers/m^2^), HD group (22 broilers/m^2^), ND-AEE group, and HD-AEE group. Each treatment group had 10 replicates, with 7 broilers per replicate in the ND and ND-AEE groups and 11 broilers per replicate in the HD and HD-AEE groups. Broiler growth performance was monitored, and hypothalamus samples were collected for transcriptome analysis on day 28. The HD group exhibited a reduced body weight (*p* < 0.01) at this timepoint compared to the ND group. However, the addition of AEE significantly improved average daily feed intake, average daily gain, and feed conversion ratio in the HD group from days 22 to 28 compared to the HD group without AEE (*p* < 0.05). The transcriptome results showed that 20 signaling pathways were commonly enriched among the groups (ND vs. HD, HD vs. HD-AEE). Several potential candidate genes were identified as involved in chicken central nervous system development and regulation of feed intake. Thus, the current study provides new insights into hypothalamic transcription patterns that are associated with the ameliorative effects of AEE in HD broilers.

## Simple Summary

High stocking density (HD) adversely affects broiler growth, and previous studies have indicated that aspirin eugenol ester (AEE) improves production performance in HD broilers; however, the specific mechanism of its feeding regulation is unknown. The main objectives of this study were to investigate the effects of AEE addition on the production performance and hypothalamic transcript levels of HD broilers under high-density rearing conditions, and to further analyze the potential link between these two factors. A group of 360 one-day-old male Arbor Acres broilers was randomly divided into four groups: an ND group (14 broilers/m^2^), HD group (22 broilers/m^2^), ND-AEE group, and HD-AEE group. AEE increased the average daily feed intake of 22–28-day-old HD broilers and decreased their feed conversion ratio. In addition, AEE upregulated the expression levels of the ingestive genes *NPY*, *AGRP*, and *GAL* mRNA in the hypothalamus of 28-day-old HD broilers. Therefore, these genes may be key factors through which AEE promotes feeding and favors growth in HD broilers. Therefore, the present study provides new insights to improve the growth and development of HD broilers.

## 1. Introduction

The importance of animal welfare and sustainable farming practices is well-established [1]. Raising broilers in a high-density (HD) stocking environment lowers production costs, makes better use of available space, and increases economic efficiency compared to those reared at normal density (ND). However, as large-scale intensive and standardized poultry farming expands, so does the density of breeding, which raises numerous problems. For example, HD environments negatively impact broiler growth [2,3], reducing production efficiency, diminishing feed intake, and increasing feed conversion ratios [4,5,6]. The impact of HD environments on animal health varies, but an increase in broiler density may be associated with higher frequencies of footpad dermatitis and scratches [7,8], reduced leg movement, and increased mortality [9]. Furthermore, HD broilers may have weakened immune systems which may induce heightened inflammatory responses and elevated levels of pro-inflammatory factors that promote physiological damage [10].

The hypothalamus is a key organ in the central nervous system that controls energy homeostasis and regulates diverse physiological functions, including feeding, body temperature, and energy metabolism [11]. The hypothalamus also secretes hormones in response to physiological needs, thereby regulating stress responses and endocrine activity. There are two main types of neurons in the hypothalamus that regulate feeding behavior: pro-feeding neurons that express neuropeptide Y (NPY)/agouti-related peptide (AGRP) and food-suppressing neurons that express proopiomelanocortin (POMC)/cocaine- and amphetamine-regulated transcript (CART) [12,13]. As the hypothalamus integrates multiple signals to influence appetite and change feed intake, and these neuropeptides play a key role in regulating appetite and energy balance, high-density feeding may affect their expression and function. However, it remains unclear how HD influences hypothalamic gene expression.

Aspirin eugenol ester (AEE) is a novel medicinal compound that is synthesized based on the principle of the pharmacological structure colocation of aspirin and eugenol [14]. AEE maintains the activity of both aspirin and eugenol but reduces the gastrointestinal irritation caused by aspirin upon ingestion and overcomes the characteristic odor and instability of eugenol without altering the clinical action of the parental compounds [15]. AEE possesses pharmacological properties that include anti-inflammatory, analgesic, anti-thrombotic, anti-atherosclerotic, anti-vascular endothelial oxidation, and antipyretic effects [15,16]. Our previous study indicated that AEE improves the production of HD broilers [5]. AEE may exert these effects by altering the transcriptional expression of key genes in the hypothalamus.

In this study, we compared the hypothalamic transcripts of ND, HD, and HD broilers that were fed AEE with the aim of investigating the effects of AEE on hypothalamic gene expression in HD broilers, as well as examining the impact of AEE on production performance. Our data provide new insights into the effects of AEE on the hypothalamus transcriptome of HD broilers.

## 2. Materials and Methods

### 2.1. Ethical Treatment

Experimental protocols were approved by the Laboratory Animal Management and Use Committee of Henan University of Science and Technology (DWFL36891-2023) on 1 October 2023. Experimental procedures were conducted in accordance with animal ethical guidelines.

### 2.2. Animals and Experimental Design

A total of 360 healthy 1-day-old Arbor Acres male broilers (Henan Quan Da Poultry Breeding Co, Ltd., Hebi, China) of similar body weight were randomly selected and fed either a basal diet or a basal diet supplemented with AEE. The birds were divided into ND and HD groups at the beginning of the study, with 14 and 22 broilers/m^2^ in the ND and HD groups, respectively, with a total of four treatment groups (ND, HD, ND-AEE, and HD-AEE). Each group comprised 10 replicates and was raised for 42 days. Chickens were fed a basal diet (Table 1) for both the starter (days 1–21) and grower (days 22–42) phases. Their diets were supplemented with AEE purchased from the Lanzhou Institute of Husbandry and Pharmaceutical Sciences of CAAS (Lanzhou, China). The concentration of AEE (0.01%) was selected based on previous studies [5] and our unpublished data. The temperature was maintained at 32 to 34 °C for the first week of the trial, and then it decreased by 1 °C every two days to 23 to 25 °C. The temperature then remained constant until the end of the trial. Housing areas were well-ventilated, and relative humidity was maintained at 55–70%. Lighting was provided for 23 h daily, with lights off for 1 h at a fixed time.

### 2.3. Growth Performance and Sample Collection

Feed intake was measured daily, and the birds were weighed at days 7, 14, 21, 28, 35, and 42 in order to determine the average daily feed intake, average daily gain, body weight, and feed conversion ratio. On days 21, 28, 35, and 42 of the experiment, 24 chickens (6 per group) were randomly selected from each of the four treatment groups. After euthanasia by cervical dislocation, their hypothalami were removed and placed on ice, promptly submerged in liquid nitrogen, and stored at −80 °C for further examination.

### 2.4. RNA Isolation and Sequencing

The Trizol method was used to extract total hypothalamic RNA. The concentration and purity of RNA were determined using a Nanodrop 2000 spectrophotometer (Thermo Fisher Scientific, Wilmington, DE, USA), and RNA integrity was detected by RNA-specific agarose electrophoresis or by using an Agilent 2100 Bioanalyzer (Agilent Technologies Inc, Santa Clara, CA, USA) (RIN scores greater than seven were considered high-quality RNA). PolyA-RNA in the total RNA was enriched using oligo (dT) magnetic beads, and random breaks were introduced to fragment the RNA to approximately 300 bp. We synthesized cDNA with reverse transcriptase using RNA as a template, followed by performing cDNA purification and PCR amplification to obtain the final library. After quality checking, the libraries were subjected to pair-end sequencing using second-generation sequencing technology on the Illumina NovaSeq 6000 platform (Illumina, San Diego, CA, USA), with a sequencing read length of 150 bp.

### 2.5. Original Data Processing, Alignment Analysis, and Quality Control

The sequencing data were transformed, examined, and statistically filtered to remove sequences, including low-quality and spliced sequences, which may have interfered with subsequent analysis. The HISAT2 (v2.1.0) software was used to align the filtered clean reads to the reference genome, obtaining the alignment efficiency. Quality control of the alignment results was performed based on gene coverage uniformity, saturation analysis, and alignment region distribution statistics.

### 2.6. Differentially Expressed Genes

Gene expression normalization was performed using FPKM based on original expression, and genes with FPKM values > 1 generally were considered as expressed genes. Gene expression was differentially analyzed using DESeq (v1.38.3) software. The conditions for screening differentially expressed genes (DEGs) were |log_2_FoldChange| ≥ 0.585 and *p* < 0.05, and the numbers were statistically compared among groups (ND vs. HD, ND vs. ND-AEE, HD vs. HD-AEE).

### 2.7. Analysis for Functional Pathway Enrichment

Gene Ontology (GO) enrichment analysis was performed using topGO (v2.50.0) to annotate the functions of the DEGs, and the results were categorized as molecular function, cellular component, and biological process. Clusterprofiler was used for Kyoto Encyclopedia of Genes and Genomes (KEGG) enrichment analysis to find significantly enriched entries of DEGs and identify the main functions they exercise.

### 2.8. Determination of Protein-to-Protein Network Interactions

The STRING database was used to determine links between genes and to visualize the network of candidate genes with Cytoscape version 3.9.1.

### 2.9. Quantitative Real-Time PCR (qRT-PCR) Analysis

Total RNA was extracted by adding hypothalamus tissue to grinding beads and lysing the cells with Trizol reagent (Invitrogen Inc., Carlsbad, CA, USA). RNA concentration and purity were assessed using a UV spectrophotometer (Agilent Technologies Inc., Santa Clara, CA, USA), and OD260/280 values in the range of 1.8–2.0 were used to indicate high purity. After the genomic DNA was removed, the RNA was reverse transcribed to cDNA using the M-MLV Reverse Transcription Kit (Accurate Bio, Changsha, Hunan, China). We added the following reagents to 20 µL of qRT-PCR reaction mixture: 2 µL of cDNA, 0.4 µL each of forward and reverse primers (Table 2), 10 µL of 2X SYBR Green Pro Taq HS Premix (Accurate Bio, Changsha, Hunan, China), and 7.2 µL of RNase-free water. The qRT-PCR reaction was carried out using the SYBR Green fluorescence method, and amplification and lysis curves were confirmed at the conclusion of the reaction. The reaction was carried out in triplicate, and the following conditions were set: an initial denaturation at 95 °C for 30 s, followed by 40 cycles of 10 s and 30 s at 95 °C and 60 °C, respectively. The qRT-PCR data were normalized using the housekeeping gene *GAPDH*. Data were analyzed using 2^−ΔΔCT^ to calculate fold change.

### 2.10. Statistical Analyses

Data were analyzed by one-way ANOVA using IBM SPSS 26.0 (SPSS Inc., Chicago, IL, USA), and the results of production performance were expressed as means ± SEM. The graphic depiction of the qRT-PCR results was performed using GraphPad Prism 9 (GraphPad Software Inc., San Diego, CA, USA). Significance between groups was assessed using Tukey’s multiple-range test; *p* < 0.05 indicated a significant difference.

## 3. Results

### 3.1. AEE Modulates the Growth Performance of HD Broilers

Broilers were reared in ND or HD environments with or without exogenous AEE in their basal diet and were assessed for changes in body weight and other parameters at intervals. Broilers in the HD group weighed significantly less (*p* < 0.05) than chickens in the ND environment at days 21, 28, 35, and 42 (Table 3). However, the body weight of chickens in the HD-AEE group at day 21 was significantly higher (*p* < 0.05) compared to the equivalent chickens without AEE. Broilers reared in HD-AEE conditions also showed a significant increase (*p* < 0.01) in average daily gain and average daily feed intake at days 22–28 relative to the HD group. Moreover, the addition of AEE to the basal diet of HD broilers significantly reduced (*p* < 0.05) the feed conversion ratio at days 22–28, 29–35, and 36–42 compared to the HD chickens. In contrast, the body weight, average daily gain, average daily feed intake, and feed conversion ratio of the broilers raised in ND and ND-AEE conditions did not differ significantly at any time. Thus, AEE specifically enhances the body weight, average daily gain, and average daily feed intake, and reduces the feed conversion ratio of broilers grown in HD environments. But the ND group still has the best feed conversion ratio compared to the ND-AEE group.

### 3.2. RNA-Sequencing Analysis

To better investigate the changes in production performance at 28 days of age, the hypothalami of 28-day-old broilers were comprehensively analyzed using high-throughput sequencing technology (Table 4). Hypothalamic sequencing results showed that an average of 54.75 million raw reads were obtained per sample. High-quality sequences accounted for >97.92% of the sequencing reads, and the percentage of bases with a base detection accuracy ≥99% ranged from 97.94 to 98.42%. In addition, the percentage of bases with a base detection accuracy ≥99.9% ranged from 94.43 to 95.73%. The total numbers of sequences from clean reads compared to the reference genome were 43,645,127 (95.50%)–56,006,660 (95.94%). Comparing the total number of sequences with only one position, the percentages were 33,698,525 (98.66%)–89,090,158 (98.46%). Finally, the total numbers of reads mapped to the gene region were compared, and the percentages were 40,102,134 (80.64%)–44,961,555 (83.07%).

### 3.3. Analysis of Differentially Expressed Genes

**Figure 1:**
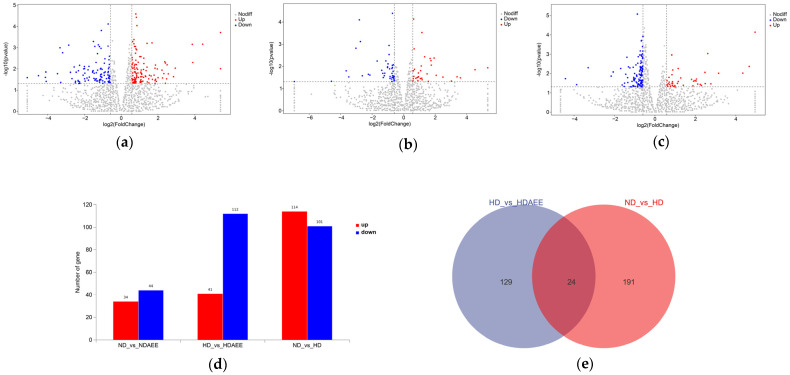
DEGs in hypothalami of broiler chickens at 28 days of age. Volcano plots of DEGs for (**a**) ND and HD groups, (**b**) ND and ND-AEE groups, and (**c**) HD and HD-AEE groups. (**d**) Number of DEGs among groups. (**e**) Venn plots of DEGs for HD group compared to ND group and HD-AEE group compared to HD group. The horizontal dashed line is the significance level threshold.

The volcano plots of the DEGs among the groups (ND vs. HD, ND vs. ND-AEE, HD vs. HD-AEE) are shown in Figure 1a–c. The expression of 215 genes differed in the transcriptomes of ND and HD groups: 114 and 101 genes were upregulated and downregulated, respectively, under HD conditions (Figure 1d). Furthermore, 153 DEGs were revealed in the hypothalami of HD and HD-AEE groups, with the upregulation of 41 genes and downregulation of 112 genes in the latter. Finally, the hypothalamic transcriptome of ND-AEE group showed 78 DEGs that were altered compared to the ND group: 34 and 44 genes were upregulated and downregulated.

According to the Venn plot results of the DEGs, 24 DEGs were common between the HD group compared to the ND group and the HD-AEE group compared to the HD group (Figure 1e). These DEGs principally involved the immune system (*SERPINB1*, *GATA3*), feeding (*POMC*, *GAL*, *MC3R*, *NPY*), neurological development (*SLC9A9*, *EN2*, *BSX*, *ISL1*, *PAX2*, *PAX6*), and growth hormone secretion (*GHRH*).

### 3.4. GO and KEGG Enrichment Analysis of Differentially Expressed Genes in Broiler Hypothalami

**Figure 2:**
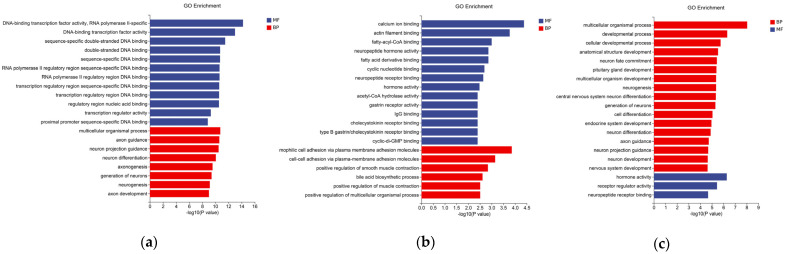
GO terms for DEGs in the hypothalami of 28-day-old broilers. Histograms of GO terms in (**a**) ND and HD groups, (**b**) ND and ND-AEE groups, and (**c**) HD and HD-AEE groups. Blue bars represent molecular functions (MFs) and red bars represent biological processes (BPs).

The DEGs that were identified in the preceding analysis were examined for GO and KEGG enrichment to better understand the biological functions of these genes. In the GO terms analysis, molecular functions and biological processes were primarily involved among groups (ND vs. HD, ND vs. ND-AEE, HD vs. HD-AEE) (Figure 2a–c). Compared with the ND group, the HD group exhibited 35 molecular functions and involvement in 218 biological processes. These molecular functions include neuropeptide receptor binding and neuropituitary hormone activity. The biological processes involved nervous system development, ingestive behavior, the modulation of hormone levels, the negative regulation of inflammation, and the modulation of glucocorticoid secretion. Compared to the HD group, the HD-AEE group exhibited 22 molecular functions and involvement in 91 biological processes. These molecular functions include hormone activity, receptor regulator activity, and neuropeptide hormone activity. The biological processes involve the development of the central nervous system, nutritional behavior, the regulation of hormone levels, and the regulation of glucocorticoid secretion.

**Figure 3:**
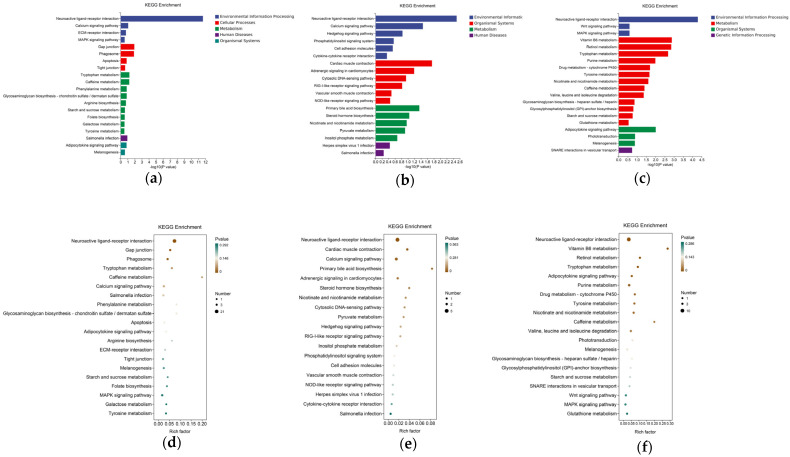
KEGG enrichment analysis of DEGs in hypothalami of 28-day-old broilers. KEGG enrichment analysis histograms of (**a**) ND and HD groups, (**b**) ND and ND-AEE groups, and (**c**) HD and HD-AEE groups. Factor plots of KEGG enrichment analysis in (**d**) ND and HD groups, (**e**) ND and ND-AEE groups, and (**f**) HD and HD-AEE groups.

The functions performed by the DEGs in the hypothalami of broilers in HD or ND environments with or without exogenous AEE were determined by KEGG enrichment analysis. The HD group was enriched in 45 signaling pathways compared to the ND group, including the adipocytokine, MAPK, Wnt, and Toll-like receptor signaling pathways. Similarly, the HD-AEE group was enriched in 35 signaling pathways compared to the HD group, including the adipocytokine, MAPK, and Wnt signaling pathways. Finally, 20 signaling pathways were commonly enriched among the groups (ND vs. HD, HD vs. HD-AEE) (Figure 3).

### 3.5. Identification of Candidate Differentially Expressed Genes and Validation of RNA-Seq Results

**Figure 4:**
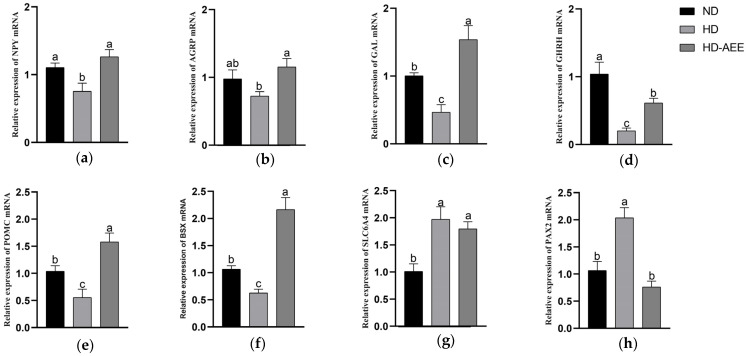
qRT-PCR validation results of DEGs. Relative mRNA expression of (**a**) *NPY*, (**b**) *AGRP*, (**c**) *GAL*, (**d**) *GHRH*, (**e**) *POMC*, (**f**) *BSX*, (**g**) *SLC6A4* and (**h**) *PAX2*. Values expressed as mean ± SEM (*n* = 6). Values with different letters are significantly different (*p* < 0.05).

**Figure 5:**
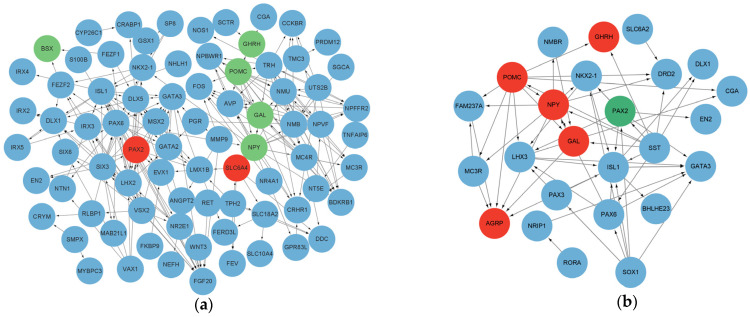
Protein network interactions between DEGs. Protein network interactions between (**a**) ND and HD groups and (**b**) HD and HD-AEE groups. Red: gene upregulation; green: gene downregulation; blue: DEGs, but not candidate genes that are predictably involved in protein networks.

Based on the GO and KEGG pathway analyses, as well as the degree of significance of the DEGs, we identified several potential candidate genes that may be involved in appetite regulation and brain development (Table 5). Eight genes (*NPY*, *AGRP*, *GAL*, *GHRH*, *POMC*, *BSX*, *SLC6A4* and *PAX2*) were selected to explore further the changes observed in the RNA-seq analysis. The qRT-PCR results of these genes exhibit similar regulatory trends to the RNA-seq results (Figure 4). Finally, protein–protein networks were further visualized, and links between potential candidate genes were established (Figure 5).

## 4. Discussion

### 4.1. AEE Modulates the Growth Performance of HD Broilers

An HD environment is a stressor that affects broiler performance [17], although results regarding the influence of stocking densities on performance are inconsistent. While HD stocking has been reported to dramatically reduce broiler performance [18,19,20,21], other data indicate that broiler performance is unaffected by stocking density [22,23]. Nevertheless, the results here support most previous studies, which highlight that HD stocking negatively impacts broiler performance. Broilers experience a decrease in average daily feed intake and average daily gain when stocking density exceeds 20 chickens/m^2^ [24]. A negative impact of stocking density on the feed conversion ratio has also been described [25]. Accordingly, the HD environment negatively affected average daily feed intake, feed conversion ratio, body weight, and average daily gain in this study. HD rearing had a greater impact on production performance during days 22–42 than during days 0–21.

We conducted an in-depth analysis of broiler production performance from days 22–42. Compared with the ND group, the HD group showed significant effects (*p* < 0.05) on average daily gain, average daily feed intake, and feed conversion ratio from days 22–28. Compared with the HD group, the HD-AEE group had a significant effect (*p* < 0.05) on the average daily feed intake, average daily gain, and feed conversion ratio of the HD broilers from days 22–28. AEE improved the production performance of the HD broilers, consistent with the findings of the previous study [5]. Interestingly, the effects of HD conditions on average daily feed intake in broilers at both days 22–28 and 29–35 were significantly lower (*p* < 0.05) than in ND chickens, whereas the addition of AEE increased (*p* < 0.05) feed intake in the HD group at days 22–28. Based on these observations, we investigated the hypothalamic transcriptome on day 28 to probe the gene expression changes involved in feeding regulation, energy metabolism, and brain development. A total of 78 DEGs (*p* < 0.05) were detected in the ND-AEE group compared to the ND group, and 153 DEGs (*p* < 0.05) were identified in the HD-AEE group compared to the HD group, which suggests a more potent effect of AEE on gene expression in the HD broiler hypothalami. As there was no discernible difference between the broiler production performances of the ND and ND-AEE groups (Table 3), we subsequently focussed on alterations in the hypothalamic transcriptome between ND and HD groups, as well as between HD and HD-AEE groups.

### 4.2. AEE Modulates Hypothalamic Feeding-Related Genes in HD Broilers

It is essential to modify animal food intake continuously to correspond with energy needs [26]. Appetite is the outcome of several pertinent regulatory factors that work in concert [12]. The control of feeding behavior by the central nervous system depends on signaling pathways that encompass key components, including core neuropeptide Y (NPY), agouti-related protein (AGRP), pro-opiomelanocortin (POMC), and cocaine- and amphetamine-regulated transcript (CART), that work in conjunction to regulate feeding behavior physiologically through an intricate interaction mechanism [27]. Feeding in poultry is encouraged particularly by the action of NPY in the hypothalamus. This neuropeptide is crucial for controlling feeding and preserving energy balance [28,29]. The expression of *NPY* mRNA is markedly increased in the hypothalamus of broiler chickens when there is a negative energy balance [30,31], and is accompanied by elevated neuronal activity [32]. NPY injected directly into brain ventricles greatly enhances and stimulates the feeding behavior of chickens [33]. The pro-feeding neuropeptide AGRP is secreted by the hypothalamus, and animals that express more AGRP consume more food while using less energy. A longer-lasting effect of increased food intake was observed when central injection of AGRP was administered instead of NPY [34]. NPY and AGRP neurons are co-expressed in the arcuate nucleus and, controlled by leptin and other factors, stimulate feeding by various modes. Melanocortin 3 receptor (MC3R) and melanocortin 4 receptor (MC4R) are blocked by AGRP [29]. The hypothalamic transcriptome analysis at day 28 here revealed that the HD group expressed lower levels (*p* < 0.05) of *NPY* than the ND group, whereas the HD-AEE group expressed higher levels (*p* < 0.05) of *NPY* and *AGRP* than the HD group. These changes in *NPY* and *AGRP* expression are congruent with variations in feed intake. Although there was no significant change, there was a tendency for the broilers in the HD environment to express hypothalamic *AGRP* at lower levels (Figure 4b). Consequently, AEE may promote broiler feeding by markedly raising *NPY* and *AGRP* expression in the HD broiler hypothalamus. In agreement with previous suggestions, brain-specific homeobox (*BSX*) expression in this study was consistent with *NPY* changes [35]. BSX may exert a regulatory role on NPY in broiler chickens. The neuropeptide NPY/AGRP controls feeding and body weight in mice when BSX is present [36]. Additionally, BSX is essential for modulating the expression of *NPY* and *AGRP* in mice [37] which influences feeding behavior and energy homeostasis. However, it is unknown why BSX exerts no effect on *POMC* expression in the hypothalamus [38]. The opposite effects of NPY/AGRP occur in neurons that express POMC/CART. POMC is a precursor polypeptide that is proteolytically processed to produce various hormones and neuropeptides, among which, the α-melanocyte-stimulating hormone (α-MSH) is an appetite suppressant. By binding to secondary neurons on MC3R and MC4R, α-MSH influences appetite and metabolism [39]. The *POMC* gene was downregulated in the hypothalamus of the HD group compared to the ND group, but was upregulated in the HD-AEE group relative to the HD group. As a result of the HD group’s reduced food intake, *POMC* changed in the opposite way from its food suppression effect. POMC has been shown to reduce appetite and cause weight loss [40]. The primary mechanism by which α-MSH regulates feeding is by activating bound MC4R [41]. Interestingly, the expression level of *MC4R* is upregulated (*p* < 0.05), contrary to the low expression of *POMC*, in low-feeding HD broilers, which may reflect competition for resources or environmental factors. Elevated *MC4R* expression also was noted following a period of food deprivation [42]. Appetite suppression also is linked to CART. Hypothalamic transcriptome data here revealed that, whereas *POMC* varied in both the ND and HD groups and in the HD and HD-AEE groups, *CART* expression was not significantly different, which may indicate that *POMC* and *CART* are not expressed exclusively in the same neurons.

Neuromedin U (*NMU*) was upregulated in the hypothalamus of 28-day-old broilers raised in the HD environment. This anorexigenic bioactive peptide is distributed widely in diverse tissues, expressed highly in the gut and brain, and plays a role in appetite regulation. Peripheral NMU also prevents ingestion by slowing stomach emptying, and may bind to NMU-R2 in the hypothalamus to suppress appetite [43]. Pharmacological studies have indicated that NMU may play a part in binge eating, and that NMU-R2 is a promising target for treating binge eating and changing eating patterns [44]. Furthermore, NMU may mediate the leptin regulation of hypothalamic–pituitary–adrenal (HPA) axis activity, thereby impacting the stress response [44]. Leptin also has a positive effect on NMU release [45]. However, only a few studies have confirmed the role of the *NMU* gene in appetite regulation in poultry. Notably, galanin and GMAP prepropeptide (*GAL*) changed in the transcriptomes, with *GAL* downregulated in the HD group compared to the ND group and upregulated in the HD-AEE group compared to the HD group. AEE may increase feed intake in HD broilers by controlling *GAL*, which is an appetitive regulator that exerts appetite-promoting effects [46]. Furthermore, GAL plays significant roles in feeding, energy regulation, and stress response, in addition to its involvement in neuroendocrine activities [47]. GAL and NPY are closely related neuroanatomically [48] and may interact to affect energy metabolism and feeding behavior [49]. Changes in the levels of the gene for growth hormone–releasing hormone (*GHRH*) in the hypothalamus transcriptomes of the HD and HD-AEE groups also may be significant. The endocrine system and hypothalamus are closely associated and regulate numerous physiological processes. Growth hormone (GH) secretion and release is stimulated by the production of GHRH by the hypothalamus. GHRH acts on the pituitary gland [50]. Although GHRH plays a critical role in the hypothalamus–pituitary–growth axis and the upregulation of *GHRH* in the HD-AEE group may have an impact on broiler growth, GH secretion is the consequence of several interrelated factors.

### 4.3. AEE Modulates Neurological Development-Related Genes in HD Broilers

Organism growth and nervous system development are interrelated processes. Serotonin transporter (SERT), which is distributed extensively throughout the central nervous system, gastrointestinal tract, and cardiovascular system, is encoded by the solute carrier family 6 member 4 (*SLC6A4*) gene [51]. By controlling 5-HT levels, SERT plays a crucial role in the central nervous system by promoting the re-uptake of the 5-hydroxytryptamine (5-HT) neurotransmitter in the synaptic gap [52]. Interestingly, 5-HT correlates strongly with mental health conditions, stress, and depression. Furthermore, 5-HT may contribute to bone formation [51] although the mechanism involved is unknown. Although it is not necessary for hypothalamus formation, BSX is crucial for the development of embryos and organs [38]. Paired box 2 (PAX2), a member of the paired box transcription factor family, primarily affects the kidney and central nervous system [53]. The deletion of the *PAX2* gene impacts behavior and reduces synaptic plasticity in mice [54]. This trial validated it at the gene level, and it may later be extended to proteomics or epigenomics for a more comprehensive understanding.

### 4.4. GO and KEGG Enrichment Analysis of Differentially Expressed Genes in Broiler Hypothalami

For a more thorough comprehension of the role and function of DEGs, we can also pursue GO and KEGG enrichment analysis. The majority of the DEGs in the hypothalamus are associated with molecular function and biological processes. More specifically, activated entries related to feeding behavior, neuropeptide hormonal activity, neurohypophyseal hormone activity, nervous system development, and cellular developmental process were observed when the ND and HD groups were compared. Activated entries pertaining to feeding behavior, central nervous system development, neuropeptide hormone activity, and endocrine hormone secretion were identified in comparisons of the hypothalamic transcriptomes of the HD and HD-AEE groups. KEGG enrichment analysis identified 36 pathways in the HD and HD-AEE groups and 46 pathways in the ND and HD groups, respectively. Their identical pathways include adipocytokine signaling, MAPK signaling, Wnt signaling, cytokine–cytokine receptor interactions, caffeine metabolism, and tryptophan metabolism. Lipocalin and leptin are key proteins produced by adipocytes in the adipocytokine pathway. Leptin plays a crucial role in controlling energy intake and metabolic rate primarily through effects on the hypothalamus, where the protein regulates neuropeptide levels [55] via JAK kinase, nuclear transcription, and STAT3 phosphorylation [56]. Lipocalin triggers MAPK, which acts to reduce blood sugar [57] and promote glucose absorption and skeletal muscle lipid oxidation [58]. Therefore, the adipocytokine signaling pathway plays a crucial part in energy metabolism. The MAPK signaling pathway also is important for numerous cellular functions, including inflammation and cell migration, differentiation, and proliferation. Leptin signaling [59], lipocalin receptor signaling [57], growth hormone signaling [60], and inflammation mediation [61] are also linked to the MAPK signaling pathway. Moreover, cell survival, proliferation, and differentiation are regulated by Wnt signaling [62], which also influences body weight and food intake and is essential for neuroendocrine regulation in the hypothalamus [63]. Our analysis and discussion demonstrated that these signaling pathways affected the experiment, changing the HD group in comparison to the ND group and the HD-AEE group in comparison to the HD group. AEE may intercalate in these pathways to enhance HD broiler growth.

## 5. Conclusions

The results of the experiment showed that broiler growth performance is negatively impacted by HD, but this effect is mitigated by supplementing their basic diet with AEE. The addition of AEE to the basal diet significantly mitigated the HD-induced decrease in the average daily feed intake of broilers during the period of 22–28 days. On day 28, it upregulated the expression levels of the feeding genes *NPY*, *AGRP*, and *GAL* in the hypothalamus of HD broilers. The modulation of expression of these genes may be the primary mechanism by which AEE encourages feeding, regulates energy metabolism, and supports HD broiler growth. Thus, the current study provides new insights into hypothalamic transcription patterns that are associated with the ameliorative effects of AEE in HD broilers and will serve as a reference for the future feeding management of poultry. In the future, we will continue to focus on the effects of HD environments on production performance and further test these research results at different levels through proteomics or epigenomics.

## Figures and Tables

**Table 1:** Composition and content of broiler basal diets.

Ingredients (%)	Starter Phase (1–21 d)	Grower Phase (22–42 d)
Corn	52.79	57.78
Soybean meal	36.89	30
Soybean oil	4	4
Wheat bran	2	2
Calcium biphosphate	1.912	1.623
Stone powder	1.222	1.171
NaCl	0.3	0.3
Choline chloride	0.3	0.26
DL-methionine	0.265	0.106
Trace element premix ^1^	0.2	0.2
L-lysine	0.038	0.045
Vitamin premix ^2^	0.03	0.03
Zea gluten meal	0	2.43
Metabolic energy(MJ/kg)	12.40	13.0
Crude protein	21.18	19.84
Lysine	1.14	1.05
Methionine	0.49	0.48
Calcium	1.02	0.85
Available P	0.45	0.42
Total P	0.69	0.63
Threonine	0.77	0.22

^1^ The trace element premix was provided with the following base mix: iron, 80 mg/Kg; copper, 10 mg/Kg; zinc, 70 mg/Kg; iodine, 0.5 mg/Kg; manganese, 80 mg/Kg; selenium, 0.3 mg/Kg. ^2^ The vitamin premixes were provided with the following base mix: Vitamin A, 9500 IU/Kg; Vitamin D3, 62.5 ug/Kg; Vitamin E, 30 IU/Kg; Vitamin K, 32.65 mg/Kg; Vitamin B1, 2 mg/Kg; Vitamin B6, 6 mg/Kg; Vitamin B12, 0.025 mg/Kg; biotin, 0.0325 mg/Kg; folic acid, 1.25 mg/Kg; pantothenic acid, 12 mg/Kg; niacin, 50 mg/Kg.

**Table 2:** Primers used for qRT-PCR analysis.

Genes	Orientation	Primer Sequence(5′ to 3′)	Length	TM ^1^	Accession Number ^2^
*GAPDH*	Forward	TGCTGCCCAGAACATCATCC	142	61	NM_204305.2
	Reverse	ACGGCAGGTCAGGTCAACAA			
*SLC6A4*	Forward	TGACAGCCACGTTCCCTTAC	114	60	XM_046903726.1
	Reverse	GGAGCTTCTGCCATTCAGGT			
*BSX*	Forward	GTCACCCCAAGCCTGAACTT	152	60	XM_046932258.1
	Reverse	TGAAAACCGAGGGGACTTCG			
*GHRH*	Forward	TGATGGACAGCCGTTACCAC	113	60	XM_015296359.4
	Reverse	GCTGGGAAACCCCTCTAACC			
*GAL*	Forward	ACTGCATCCGTGGGACATTT	110	60	NM_001145389.3
	Reverse	CCCACACACCTCTGCATCTT			
*POMC*	Forward	GAGAACAGCAAGTGCCAGGA	146	60	NM_001398117.1
	Reverse	ACGTACTTGCGGATGCTCTC			
*NPY*	Forward	CCTTCGATGTGGTGATGGGA	106	59	NM_205473.2
	Reverse	ATGCACTGGGAATGACGCTA			
*AGRP*	Forward	ATTGCACCGACGAACTTTGC	97	60	XM_025154207.3
	Reverse	GCTGGCAAGAAATGAACGCA			
*PAX2*	Forward	GGCGAGAAGAGGAAACGTGA	94	60	XM_025151472.3
	Reverse	GAAGGTGCTTCCGCAAACTG			

^1^ TM, Melting Temperature. ^2^ Accession number refers to GenBank (NCBI).

**Table 3:** Production performance of broilers.

Parameter	Day	Experimental Group ^1^				*p*-Value
		ND	HD	ND-AEE	HD-AEE	
Body weight (g)	7	165.29 ± 4.07	166.40 ± 1.02	159.78 ± 3.74	162.00 ± 3.03	0.461
	14	455.40 ± 2.48	453.14 ± 1.48	454.51 ± 3.94	453.72 ± 3.73	0.958
	21	991.71 ± 8.08 ^a^	964.17 ± 6.52 ^b^	978.60 ± 6.26 ^ab^	992.75 ± 4.67 ^a^	0.022
	28	1632.40 ± 24.72 ^a^	1542.47 ± 5.83 ^b^	1632.90 ± 15.59 ^a^	1593.33 ± 18.89 ^ab^	0.007
	35	2372.03 ± 38.85 ^a^	2113.58 ± 25.82 ^b^	2416.33 ± 38.79 ^a^	2182.35 ± 26.38 ^b^	<0.001
	42	3006.84 ± 90.18 ^a^	2511.22 ± 23.87 ^b^	3049.47 ± 60.57 ^a^	2647.44 ± 46.22 ^b^	<0.001
Average daily gain (g)	1–7	18.28 ± 0.57	18.37 ± 0.14	17.43 ± 0.54	17.81 ± 0.43	0.449
	8–14	41.44 ± 0.34	40.96 ± 0.17	42.10 ± 0.55	41.67 ± 0.51	0.317
	15–21	76.62 ± 1.13 ^a^	72.97 ± 1.00 ^b^	74.87 ± 1.36 ^ab^	75.73 ± 0.90 ^ab^	0.159
	22–28	92.99 ± 1.76 ^a^	85.02 ± 2.35 ^b^	94.80 ± 1.09 ^a^	92.65 ± 0.95 ^a^	0.003
	29–35	106.49 ± 1.86 ^a^	78.59 ± 1.41 ^b^	111.08 ± 3.58 ^a^	84.76 ± 1.28 ^b^	<0.001
	36–42	93.63 ± 4.46 ^a^	56.80 ± 2.99 ^c^	93.04 ± 2.09 ^a^	66.12 ± 2.37 ^b^	<0.001
	1–42	72.01 ± 1.19 ^a^	58.79 ± 0.79 ^c^	71.83 ± 0.73 ^a^	62.64 ± 0.90 ^b^	<0.001
Average daily feed intake (g)	1–7	20.29 ± 0.60	20.12 ± 0.25	19.67 ± 0.31	20.25 ± 0.13	0.630
	8–14	50.58 ± 0.34	50.42 ± 0.07	51.74 ± 0.85	50.63 ± 0.28	0.231
	15–21	93.41 ± 1.93	90.45 ± 0.29	91.55 ± 1.25	92.34 ± 0.25	0.358
	22–28	124.77 ± 1.13 ^a^	119.63 ± 0.85 ^b^	125.27 ± 1.17 ^a^	123.11 ± 0.82 ^a^	0.005
	29–35	178.71 ± 3.74 ^a^	145.41 ± 1.15 ^b^	177.12 ± 4.08 ^a^	149.68 ± 1.17 ^b^	<0.001
	36–42	158.11 ± 6.59 ^ab^	145.34 ± 2.53 ^b^	166.37 ± 3.99 ^a^	148.23 ± 0.82 ^b^	0.008
	1–42	104.31 ± 1.77 ^a^	95.23 ± 0.67 ^b^	105.77 ± 1.30 ^a^	97.72 ± 0.44 ^b^	<0.001
Feed conversion ratio (g/g)	1–7	1.11 ± 0.02	1.10 ± 0.02	1.13 ± 0.02	1.14 ± 0.02	0.490
	8–14	1.22 ± 0.01	1.23 ± 0.004	1.23 ± 0.01	1.22 ± 0.01	0.741
	15–21	1.22 ± 0.01	1.24 ± 0.02	1.22 ± 0.01	1.22 ± 0.02	0.666
	22–28	1.35 ± 0.03 ^b^	1.44 ± 0.02 ^a^	1.33 ± 0.02 ^b^	1.35 ± 0.03 ^b^	0.048
	29–35	1.70 ± 0.01 ^c^	1.85 ± 0.02 ^a^	1.67 ± 0.02 ^c^	1.79 ± 0.01 ^b^	<0.001
	36–42	1.77 ± 0.02 ^c^	2.58 ± 0.13 ^a^	1.75 ± 0.04 ^c^	2.24 ± 0.05 ^b^	<0.001
	1–42	1.45 ± 0.02 ^c^	1.62 ± 0.02 ^a^	1.47 ± 0.01 ^c^	1.56 ± 0.02 ^b^	<0.001

^1^ ND, normal stocking density + basal diet; HD, high stocking density + basal diet; ND-AEE, normal stocking density + basal diet + AEE (0.01%); HD-AEE, high stocking density + basal diet + AEE (0.01%). ^a, b, c^ Values with different letters are significantly different (*p* < 0.05).

**Table 4:** Assessment of transcriptome quality.

Groups ^1^	Raw Reads No.	Clean Reads No.	Clean Reads (%)	Q20 (%)	Q30 (%)	Total Mapped	Uniquely Mapped	Mapped to Gene
ND	54,627,494	53,590,940	98.10	98.42	95.73	51,313,726 (95.75%)	50,590,855 (98.59%)	41,199,424 (81.44%)
ND	59,561,068	58,377,802	98.01	98.41	95.71	56,006,660 (95.94%)	55,201,661 (98.56%)	45,369,218 (82.19%)
ND	38,935,978	38,159,888	98.01	98.34	95.50	36,499,616 (95.65%)	36,000,012 (98.63%)	29,367,686 (81.58%)
ND	53,717,526	52,641,386	98.00	98.35	95.57	50,426,148 (95.79%)	49,731,343 (98.62%)	40,102,134 (80.64%)
HD	55,126,948	54,012,172	97.98	98.24	95.33	51,716,515 (95.75%)	50,960,061 (98.54%)	41,851,625 (82.13%)
HD	57,971,320	56,861,700	98.09	98.33	95.55	54,397,081 (95.67%)	53,605,331 (98.54%)	43,806,353 (81.72%)
HD	52,914,462	51,848,830	97.99	98.30	95.47	49,638,355 (95.74%)	48,907,266 (98.53%)	39,821,221 (81.42%)
HD	52,405,628	51,378,738	98.04	98.30	95.45	49,270,482 (95.90%)	48,560,595 (98.56%)	39,948,204 (82.26%)
ND-AEE	96,277,166	94,644,780	98.30	97.94	94.43	90,487,299 (95.61%)	89,090,158 (98.46%)	72,843,917 (81.76%)
ND-AEE	59,467,550	58,389,396	98.19	98.07	94.80	55,852,055 (95.65%)	55,057,385 (98.58%)	44,605,943 (81.02%)
ND-AEE	36,419,636	35,667,052	97.93	98.32	95.51	34,156,642 (95.77%)	33,698,525 (98.66%)	27,414,297 (81.35%)
ND-AEE	59,226,338	58,023,966	97.97	98.25	95.34	55,618,180 (95.85%)	54,830,328 (98.58%)	45,123,700 (82.30%)
HD-AEE	42,148,190	41,288,004	97.96	98.25	95.35	39,459,283 (95.57%)	38,890,828 (98.56%)	32,095,712 (82.53%)
HD-AEE	52,143,530	51,121,882	98.04	98.28	95.41	48,840,492 (95.54%)	48,123,218 (98.53%)	39,620,219 (82.33%)
HD-AEE	58,649,914	57,427,750	97.92	98.22	95.27	54,935,238 (95.66%)	54,125,547 (98.53%)	44,961,555 (83.07%)
HD-AEE	46,643,014	45,702,484	97.98	98.26	95.36	43,645,127 (95.50%)	42,994,732 (98.51%)	35,145,550 (81.74%)

^1^ ND, normal stocking density + basal diet; HD, high stocking density + basal diet; ND-AEE, normal stocking density + basal diet + 0.01%AEE; HD-AEE, high stocking density group + basal diet + 0.01% AEE.

**Table 5:** Candidate genes related to appetite regulation and brain development.

Groups	Expression Type	Genes	Description	Log_2_ Fold Change	*p*-Value	GO Terms	KEGG Terms
ND vs. HD	Upregulated	*PAX2*	paired box 2	1.35859670192997	0.012364407545465	Anatomical structure development	
		*SLC6A4*	solute carrier family 6 member 4	2.32591701032793	0.0168377568709956	Brain development; central nervous system development	
		*NMU*	neuromedin U	2.84911998315659	0.0235247051040035	Neuropeptide receptor binding; feeding behavior	Neuroactive ligand–receptor interaction
	Downregulated	*BSX*	brain specific homeobox	−2.84526873595193	0.000768191298709178	Nervous system development; brain development; feeding behavior	
		*GHRH*	growth hormone–releasing hormone	−1.79719156328328	0.0112923940240136	Hormone activity	
		*GAL*	galanin and GAMP prepropeptide	−1.96984201020936	0.0159764943487804	Nervous system development; feeding behavior	Neuroactive ligand–receptor interaction
		*POMC*	proopiomelanocortin	−4.11476242453669	0.0253199901809521	Neuropeptide receptor binding; hormone activity	Neuroactive ligand–receptor interaction; adipocytokine signaling pathway; melanogenesis
		*NPY*	neuropeptide Y	−1.3453977903136	0.0296508934963984	Neuron differentiation; feeding behavior	Neuroactive ligand–receptor interaction; adipocytokine signaling pathway
HD vs. HD–AEE	Upregulated	*POMC*	proopiomelanocortin	4.95937427051282	0.0000731210766572	Neuropeptide receptor binding; melanocortin receptor binding	Neuroactive ligand–receptor interaction; adipocytokine signaling pathway; melanogenesis
		*AGRP*	agouti–related peptide	4.67024721779006	0.00437397674819453	Feeding behavior; melanocortin receptor binding	Adipocytokine signaling pathway
		*GAL*	galanin and GMAP prepropetide	2.48206229418743	0.00885532689389276	Feeding behavior; neuropeptide receptor binding	Neuroactive ligand–receptor interaction; adipocytokine signaling pathway; melanogenesis
		*BSX*	brain–specific homeobox	3.15582202791072	0.00971339458154058	Nervous system development; feeding behavior	
		*NPY*	neuropeptide Y	0.990330132410699	0.0430652490292915	Feeding behavior; nervous system development	Neuroactive ligand–receptor interaction; adipocytokine signaling pathway
		*GHRH*	growth hormone–releasing hormone	1.88699917744512	0.0444791550523927	Receptor regulator activity; signaling receptor binding	
	Downregulated	*PAX2*	paired box 2	−1.21653626578342	0.0496875384577193	Animal organ development; biological regulation	

Abbreviations: GO, Gene Ontology; KEGG, Kyoto Encyclopedia of Genes and Genomes.

## Data Availability

The corresponding author can provide access to the collected and analyzed data sets from this study upon request.

## Abbreviations

The following abbreviations are used in this manuscript:

HD	high stocking density
ND	normal stocking density
AEE	aspirin eugenol esters
DEGs	differential expression genes
MC3R	melanocortin 3 receptor
MC4R	melanocortin 4 receptor
PAX6	paired box 6
α-MSH	alpha-melanocyte-stimulating hormone
NMU	neuromedin U
GH	growth hormone
SERT	serotonin transporter
5-HT	5-hydroxytryptamine
SERPINB1	serpin family B member 1
GATA3	gata binding protein 3
SLC9A9	solute carrier family 9 member A9
EN2	engrailed homeobox 2
ISL1	isl lim homeobox 1
PAX2	paired box 2
SLC6A4	solute carrier family 6 member 4
BSX	Brain-specific homeobox
GHRH	growth hormone–releasing hormone
GAL	galanin and GAMP prepropeptide
POMC	proopiomelanocortin
AGRP	agouti-related peptide
NPY	neuropeptide Y
CART	cocaine- and amphetamine-regulated transcript
KEGG	Kyoto Encyclopedia of Genes and Genomes
GO	Gene Ontology
